# The Latest Theory of Sea-Sickness

**Published:** 1883-07

**Authors:** 


### THE LATEST THEORY OF SEA-SICKNESS.

Perhaps the most acceptable theory to-day is the one which places the origin of the trouble in the inner ear. The ear consists of three parts : the outer of these runs in as far as the drum ; the middle part is inside the drum, and contains the chain of ear-bones ; while the inner ear is a complicated affair, containing the essential organ of hearing.

As far as we are concerned, the inner ear is a membranous bag filled with fluid, and situated in the solid bone. From the back part of this bag run out three semicircular tubes communicating at both their ends with the bag or vestibule. These run in three different planes, and are lined with hair-like nerve-filaments, which are much more abundant and more sensitive at the anterior part of the tubes. The tubes are filled with liquid in which float little calcareous particles, the otoliths. These tubes are known as the semicircular canals. It was difficult to see what connection with the sense of hearing these canals could possibly have, and some time ago it was noticed that injuries to these impaired the sense of hearing in no way, but caused most curious effects in the loss of equilibrium.—*From* " *The Cause of Sea-Sickness,*" *by* R. W. Lovett, *in Popular Science Monthly for July.*

